# Midline shift in relation to thickness of traumatic acute subdural hematoma predicts mortality

**DOI:** 10.1186/s12883-015-0479-x

**Published:** 2015-10-24

**Authors:** Ronald HMA Bartels, Frederick JA Meijer, Hans van der Hoeven, Michael Edwards, Mathias Prokop

**Affiliations:** Department of Neurosurgery, Radboud University Medical Center, Nijmegen, The Netherlands; Department of Radiology, Radboud University Medical Center, Nijmegen, The Netherlands; Department of Intensive Care Medicine, Radboud University Medical Center, Nijmegen, The Netherlands; Department of Trauma and Emergency Surgery, Radboud University Medical Center, Nijmegen, The Netherlands

**Keywords:** Acute subdural hematoma, Survival, Prediction, CT

## Abstract

**Background:**

Traumatic acute subdural hematoma has a high mortality despite intensive treatment. Despite the existence of several prediction models, it is very hard to predict an outcome. We investigated whether a specific combination of initial head CT-scan findings is a factor in predicting outcome, especially non-survival.

**Methods:**

We retrospectively studied admission head CT scans of all adult patients referred for a traumatic acute subdural hematoma between April 2009 and April 2013. Chart review was performed for every included patient. Midline shift and thickness of the hematoma were measured by two independent observers. The difference between midline shift and thickness of the hematoma was calculated. These differences were correlated with outcome. IRB has approved the study.

**Results:**

A total of 59 patients were included, of whom 29 died. We found a strong correlation between a midline shift exceeding the thickness of the hematoma by 3 mm or more, and subsequent mortality. For each evaluation, specificity was 1.0 (95 % CI: 0.85–1 for all evaluations), positive predictive value 1.0 (95 % CI between 0.31–1 and 0.56–1), while sensitivity ranged from 0.1 to 0.23 (95 % CI between 0.08–0.39 and 0.17–0.43), and negative predictive value varied from 0.52 to 0.56 (95 % CI between 0.38–0.65 and 0.41–0.69).

**Conclusions:**

In case of a traumatic acute subdural hematoma, a difference between the midline shift and the thickness of the hematoma ≥ 3 mm at the initial CT predicted mortality in all cases. This is the first time that such a strong correlation was reported. Especially for the future development of prediction models, the relation between midline shift and thickness of the hematoma could be included as a separate factor.

## Background

An acute subdural hematoma (ASDH) is a devastating clinical entity with a clinical outcome that is difficult to predict clinical ranging from completely independent functioning and death. Mortality rates up to 60 % have been published [1]. A major dilemma is whether or not to institute maximal treatment or abstain from further life saving measures, as these may prove to be futile. Although trauma patients often sustain multiple injuries, the prediction of survival appears to be largely dependent on the extent of the intracranial abnormality. Multiple prediction models have been developed. Most frequently, these models do not predict mortality, but rather try to differentiate between the potential for a good clinical outcome versus disability, vegetative state or death [1–4]. However, the validity of most prediction models has proven to be suboptimal [5]. The CRASH and IMPACT prediction model included midline shift (MLS) but not the difference between MLS and the thickness of an possible hematoma [6]. The concept of brain swelling is not new [7], but has not been incorporated into the current prediction models. Most models focus mainly on MLS as a separate entity [8]. MLS however is caused by the hematoma itself and the concomitant brain edema. The effect of the brain swelling can be shown on the initial head CT scan by the relationship of the thickness of the hematoma and the midline shift (MLS). If the MLS exceeds the thickness of the hematoma, then brain swelling should be supposed. The MLS in relation to the thickness of the hematoma has not explicitly been included in most models. We hypothesize that the correlation between the magnitude of MLS and thickness of the hematoma is a predictor of mortality and should be incorporated as such in future prognostic models.

**Fig. 1:**
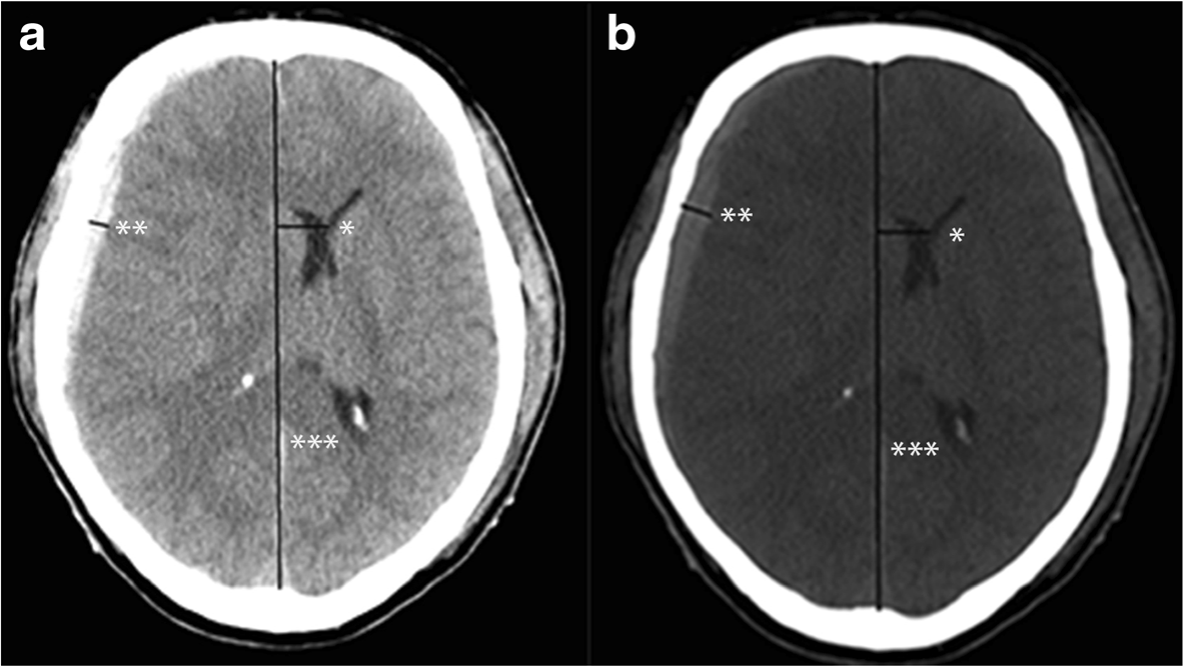
Depicting a CT scan of a patient who suffered from a right sided acute subdural hematoma. The standard windows W/L is shown in (**a**), and also the method to measure the thickness of the hematoma (**) and the midlineshift (midline ***, shift *). The thickness of the hematoma was 5 mm and the MLS 15 mm. After adapting the windows W/L was to the suggested level (**b**) the thickness of the hematoma was 10 mm and the MLS 15 mm

## Methods

The Radboud University Medical Center is a level I trauma center and approximately 400 multiple trauma patients with an Injury Severity Score (ISS) of more than 16 are admitted annually. All adult patients with a traumatic ASDH between April 2009 and April 2013 whom were offered neurosurgical treatment (evacuation hematoma) were included. The data of patients including concomitant diseases, presence of hemorrhagic diathesis and ISS, as well as the CT scans at admission were retrospectively retrieved from an electronic hospital database.

All patients were offered standard maximal intensive care treatment including ICP monitoring, hyperosmolar treatment, sedation, induced hypothermia, and decompressive surgery (evacuation of the hematoma without replacement of the bone flap except when then brain was very swollen, removal of the bone flap was considered when the intracranial pressure raised irrespective of conservative methods).

Two blinded observers (RB, FM) evaluated twice, with one month interval, head CT scans which were part of the patient’s electronic database. Measurements of MLS and the thickness of the hematoma were obtained at the level of the frontal horns using the following protocol: on the initial CT scan (viewer: Agfa Impax version 6.4, Ortsel, Belgium), MLS was measured at the level of the frontal horns using standard window widths (WW) and window levels (WL) to evaluate brain parenchyma (WW 86, WL 30). MLS was defined as the displacement of the septum pellucidum in relation to the midline in millimeters [2]. The subdural hematoma was evaluated with adjusted WW and WL to minimize underestimation of hematoma thickness (WW 300, WL 120). The reason for choosing this method was that after preliminary review of some CT scans, the TH could reliably be established, and more importantly the midline could clearly be defined (Fig. 1). The level of the frontal horns was used for the measurement of the TH since, in our opinion, the impact on the brain will be expressed by a MLS exceeding the TH at the same level. Furthermore it will reduce inter-rater variability. The observers were blinded to the final outcome.

The difference between the MLS and the thickness of the hematoma was expressed in millimeters (mm). Then 1 mm, 2 mm and 3 mm respectively were added to the actual thickness of the hematoma, and the difference with the MLS for each new situation obtained.

So, at the end four differences were compared, the actual difference: MLS minus thickness of the hematoma, and three in which 1 mm, 2 mm and 3 mm respectively were added to the thickness of the hematoma. The difference was expressed as a nominal value (MLS more than thickness hematoma or not), and correlated with clinical outcome.

The work described has been carried out in accordance with The Code of Ethics of the World Medical Association (Declaration of Helsinki) for experiments involving humans. The study has been approved by the institutional review board CMO Arnhem - Nijmegen (IRB). The IRB waived informed consent due to the nature of the investigations.

For statistical analysis SPPS 20 (Armonk, NY, USA: IBM Corp) was used. For intra – and interobserver reliability Cohen’s kappa’s coefficient was used. The kappa’s coefficient was graded as follows: below 0.20, poor agreement; between 0.21 and 0.4, fair agreement; between 0.41 and 0.60 moderate agreement, between 0.61 and 0.80 good agreement; and > 0.80 perfect agreement [9]. For correlation between outcome expressed as survivor or non – survivor and the difference between MLS and TH, Cramer’s V coefficient was used. Sensitivity, specificity, positive predictive value (PPV), and negative predictive value (NPV) were calculated as were the respective 95 % confidence intervals (95 % CI). For comparison of categorical data the Chi square test was used, and for independent numerical data the independent student-*t* test. Statistical significance was assumed for *p* <0.05. Data are represented as mean ± standard deviation (range) or as median (total range) depending on the distribution of the data. This study conforms to the Strengthening the Reporting of Observational studies in Epidemiology (STROBE) guidelines.

## Results

Fifty-nine patients were included. The mean age was 62.7 ± 18.7 years (19.2–87.9). A total of 51.7 % of the patients had an initial GCS score of five or less before intubation. At discharge 12 (20.3 %) patients made a good neurologic recovery, 10 (16.9 %) had a neurologic deficit but were independently functioning at home, and 7 (11.9 %) patients required admission to a nursing home. Thirty (50.8 %) patients did not survive. Cerebral trauma was deemed the major cause of death in all patients. Their median survival was 2 days (0–276). Six patients survived for less than one day. Of the non-surviving patients, 21 (70 %) had an initial GCS score of five or less. None of these patients left the hospital after the accident. Patients were not excluded for this analysis. In Tables 1 and 2 concomitant diseases were represented, respectively the presence of a bleeding diathesis. These were grouped according to a dichotomized outcome (dead or alive). For both investigated items a statistically significant difference was not found (*p* = 0.908 respectively *p* = 0.901). The mean ISS for surviving patients was 24.6 ± 11.9, and for non-surviving patients 28.5 ± 11.9. This difference did not reach statistical difference. All patients except for one underwent surgical evacuation of hematoma. One patient had a very bad clinical situation, and further therapeutic options were considered as not meaningful to survival. This patient did not belong to the group of patients whose MLS exceeded the thickness of the hematoma plus 3 mm.

**Table 1:** Concomittant disease and relation to dichotomized outcome (survived versus dead)

Concomittant disease	Survived	Dead	Total
None	7	10	17
Hypertension	3	1	4
Diabetes mellitus	2	3	51
Occlusive vascular disease	11	14	25
Psychiatric disorder	1	0	1
Alcohol abuse (>5E daily)	1	1	2
Various (e.g. Downs’ syndrome)	4	1	6
Total	29	30	59

**Table 2:** Bleeding diathesis and relation to dichotomized outcome (survived versus dead)

Bleeding diathesis	Survived	Dead	Total
None	18	16	34
Alcohol abuse (>5E daily)	1	1	2
Acetylsalicylic acid	6	7	13
Vitamin-K antagonist	4	6	10
Total	29	30	59

The intra – and inter- rater reliability is represented in Table 3. A very strong correlation was found between MLS and thickness of hematoma plus 3 mm (Table 4). All patients (*N* = 8) in whom the initial CT scan showed an MLS that was 3 mm greater (mean 4.7 mm, range 3.1–7.4 mm) than the maximal thickness of the hematoma died. Except for one patient the initial GCS score was reported 3. The other patient had an GCS score of 6. Median survival was six days (0–276 days). The risk of 14 day mortality according to CRASH [6] for these patients was 66.4 % (48.6 % – 84.5 %) and risk for unfavourable outcome (dead or severe disability at six months was 89.9 % (85.4 % – 95.6 %). The mean mortality rates corresponding to the respective measurements of the observers if the MLS excessed the TH, the TH + 1 mm, and finally the TH + 2 mm were respectively 75, 83.4, and 81.8 %.

Sensitivity, specificity, positive predictive value (PPV), and negative predictive value (NPV) of MLS in relationship to thickness of hematoma plus 3 mm were calculated for each evaluation session for both observers (Table 5). In all evaluations the specificity was 1.0 (95 % CI in all evaluations 0.85–1.0) and PPV was 1.0 (with varying 95 % CI from 0.38–0.65 till 0.41–0.69). Sensitivity differed from 0.1 (95 % CI: 0.03–0.28) till 0.23 (95 % CI: 0.17–0.43), and the NPF from 10 0.52 (95 % CI: 0.38–0.65) till 0.56 (95 % CI: 0.41–0.69).

**Table 3:** Intra – and inter- observer reliability of the two evaluations of initial CT (MLS: midline shift, TH: thickness hematoma) In the second through last column 1, 2 respectively 3 mm were added to TH

	MLS > TH	MLS > TH + 1	MLS > TH + 2	MLS > TH +3
Intra-rater reliability Observer 1	0.68 ± 0.12	0.3 ± 0.15	0.42 ± 0.17	0.64 ± 0.19
Intra-rater reliability Observer 2	0.62 ± 0.11	0.73 ± 0.11	0.71 ± 0.12	0.82 ± 0.13
Interobserver First evaluation	0.67 ± 0.11	0.62 ± 0.13	0.64 ± 0.14	0.74 ± 0.14
Interobserver Second evaluation	0.40 ± 0.12	0.30 ± 0.15	0.38 ± 0.16	0.73 ± 0.18

**Table 4:** Correlation between difference in midline shift and thickness hematoma + 3 mm with outcome. Eight patients were included totally

	Cramer’s V	*p*-value	Number of patients
Observer 1 First evaluation	0.331	0.01	6
Observer 1 Second evaluation	0.228	0.08	3
Observer 2 First evaluation	0.361	0.006	7
Observer 2 Second evaluation	0.299	0.02	5

**Table 5:** Sensitivity, specificity, PPV and NPV for each evaluation session and each observer when comparing MLS to thickness of the hematoma plus 3 mm [value (95 % confidence interval)]

	Observer 1		Observer 2	
Evaluation	1	2	1	2
Sensitivity	0.2 (0.08–0.39)	0.1 (0.03–0.28)	0.23 (0.17–0.43)	0.17 (0.06–0.35)
Specificity	1 (0.85–1)	1 (0.85–1)	1 (0.85–1)	1 (0.85–1)
PPV	1 (0.52–1)	1 (0.31–1)	1 (0.56–1)	1 (0.46–1)
NPV	0.55 (0.41–0.68)	0.52 (0.38–0.65)	0.56 (0.41–0.69)	0.53 (0.4–0.67)

## Discussion

This study clearly showed that the value of MLS in relation to the thickness of the traumatic ASDH needs to be re-considered. In fact, if measurements were made in accordance with our protocol, the relationship between MLS and thickness of the hematoma plus 3 mm was an absolute predictor of death. In these patients, it appeared that the trauma resulted in more damage than just an ASDH. Indeed, it also disrupted normal brain anatomy and physiology resulting in very rapid and significant swelling. This in turn is reflected in the poor neurological condition (comatose or already sedated, intubated, and ventilated) noted at admission. As such, one would anticipate that a correlation would exist between the difference of MLS and thickness of the hematoma, and clinical presentation. This should be taken into account when developing prediction models, since modern ones as CRASH and IMPACT did include the MLS but not the relationship between MLS and thickness of the hematoma [6].

The correlation, which proved to be very evident in this report was not as compelling in the study by Zumkeller et al. [7]. The relationship between MLS and thickness of the hematoma was described (they used the term brain swelling factor). However, a clear cut-off point as in our study was not found. The explanation could be threefold: 1) their CT scans were not available in a digital format such that measurements were manually done without magnification (current images are bigger than on the hardcopies with 12 images on one page); 2) because of the fact that the CT scans were not digitally available the window level and window width could not be adapted to a uniform level more clearly depicting the difference between hematoma and the bone, and therefore measurement of the actual thickness of the hematoma could be incorrect; 3) lack of standardization in measuring MLS and the thickness of the hematoma. It would not have been possible to correct the window width level of the scans at that time. The lack of a defined protocol for measuring MLS and SDH size on CT scans certainly contributes to a lesser degree of inter-rater reliability, which in turn diminishes the power to predict outcome in the currently available models. It might be very appealing to compare the volume of the hematoma with the MLS. However, in our opinion, it would not be correct to compare the volume of the subdural hematoma to the MLS estimated at one slide at a single level. Measuring the total volume of brain shift would be more appropriate. However, we are not aware of a CT algorithm that can estimate this variable. Prediction models comparing volume of the hematoma to the MLS (measured at a single level at one slide) have not been able to demonstrate a clear correlation with mortality.

Furthermore, the relationship between MLS and thickness of the hematoma was proven using specified CT window level setting and definition of MLS. Indeed, when brain tissue settings were used to measure the hematoma, an accurate representation of the clot was not obtained and thickness was noted to be decreased. Therefore, the window-level settings of the head CT should be standardized. Very recently Maas and co-authors pleaded for standardization of data collection in order to improve research in traumatic brain injury. It was remarkable that their plea was really restricted to the construction of the database [10]. Standardization of the settings of the CT was not mentioned. Our study has several limitations. Since the analysis was performed in exclusively adult patients, the results can not be extrapolated on pediatric population. We used a retrospective design. However, we think that both the measurements and the endpoint of the study (mortality) were simple, easy to retrieve and very robust. More importantly, a prospective design could raise important ethical issues in which over - and under treatment would be possible to fulfill the hypothesis. In our institute MLS by itself has never been a parameter to start or to withhold surgical therapy. However, a prospective study would be feasible if the a comparison could be made between the initially predicted outcome and the virtual one. It must be obligatory that the predicted outcome may not interfere with the provided treatment. We did neither correct for severity of potential multiple injuries. However, for this study we purposed to address explicit attention to the possible predictive value of the relationship between MLS and thickness of the hematoma, and the high specificity, we think that additional analyses will not contribute to the results. Thus, we think, that additional analyses will obscure the main message. For future studies, however, the more extensive analyses in larger populations are mandatory.

The sample size of our study is relatively small, which is reflected by 95 % CI’s. However, the values for sensitivity, specificity, PPV and NPV are very similar as are the widths of the confidence intervals suggesting reliability of the results. Further larger studies are therefore necessary to confirm these findings. Because of the size of this sample size, we did not investigate the added predictive value in existing prediction models as CRASH and IMPACT. Neither did we perform multi-variate analysis including more known predictors, since the results would be certainly flawed by the sample size.

Again we would like the stress that the difference between MLS and thickness of the hematoma to which 3 mm is added is an important predictor for mortality and could be considered to be included in prediction models that are adapted or developed in the future. We think that this factor would be stronger predictor for outcome than midline shift, thickness of the hematoma and/or volume of the hematoma as a separate predictive factor. Especially, since in the current models a difference between these values is not taken into account. If our result is confirmed in larger studies, we think that it could even act as a ‘threshold’ marker predicting a 100 % mortality. At this moment, we feel that any speculation about the consequences of such a strong marker is not justified.

## Conclusions

In patients with a traumatic ASDH a clear correlation between MLS and thickness of the hematoma was found. All patients with MLS exceeding the thickness of the hematoma by 3 mm or more died. In our opinion, it is the first time that such a strong correlation has been reported. The main purpose of this study was to draw attention to this relation that could considered to be incorporated as a factor in future prognostic models. It should not be used as a standalone predictive factor!

It should however clearly be stated that MLS is to be determined using the CT window levels provided in this paper. A uniform definition of grading and a standardized method for measurement on head CT scan should also be applied to all future prediction models.

## Abbreviations

ASDH: Acute subdural hematoma
CI: Confidence interval
ISS: Injury severity score
Mm: Millimetres
MLS: Midline shift
NPV: Negative predictive value
PPV: Positive predictive value
